# Stat3 accelerates Myc induced tumor formation while reducing growth rate in a mouse model of breast cancer

**DOI:** 10.18632/oncotarget.11667

**Published:** 2016-08-29

**Authors:** Jing-Ru Jhan, Eran R. Andrechek

**Affiliations:** ^1^ Department of Physiology, Michigan State University, East Lansing, MI 48824, USA

**Keywords:** breast cancer, mammary gland, Myc, Stat3, tumor latency

## Abstract

Elevated Myc expression has been noted in basal breast cancer but therapies targeting Myc directly are lacking. It is therefore critical to characterize the interaction of Myc with other genes and pathways to uncover future potential therapeutic strategies. In this study, we bioinformatically predicted a role for Stat3 in Myc induced mammary tumors and tested it using mouse models. During normal mammary function, loss of Stat3 in Myc transgenic dams resulted in lethality of pups due to lactation deficiencies. We also observed that deletion of Stat3 in the mammary glands of MMTV-Myc mice unexpectedly resulted in increased and earlier hyperplasia and expedited tumorigenesis. However, despite arising earlier, Myc tumors lacking Stat3 grew more slowly with alterations in the resulting histological subtypes, including a dramatic increase in EMT-like tumors. We also observed that these tumors had impaired angiogenesis and a slight decrease in lung metastases. This metastatic finding is distinct from previously published findings in both MMTV-Neu and MMTV-PyMT mouse models. Together, the literature and our current research demonstrate that Stat3 can function as an oncogene or as a tumor repressor depending on the oncogenic driver and developmental context.

## INTRODUCTION

Amplification and overexpression of Myc has been noted in a high percentage of breast cancers, 22% by mRNA and up to 45% by protein [1–3]. Elevated Myc expression is most commonly noted in basal breast cancer, the most aggressive molecular subtype [1, 2]. Numerous *in vitro* studies have demonstrated that Myc acts as an oncogenic transcription factor, involved in cell cycle, proliferation [3], apoptosis, genomic instability [4] and angiogenesis [5]. A causative role for Myc in breast cancer was demonstrated by overexpressing Myc in transgenic mouse models [6, 7]. While overexpression of Myc in the mammary gland resulted in tumors after a long latency, tumors were not noted to be highly metastatic [8]. Expression of Myc is controlled through both post-translational modification and transcriptional regulation. Notably, one of the transcriptional factors regulating mRNA levels of Myc is Stat3 (Signal transducer and activator of transcription 3) [9].

Stat3 is constitutively activated in breast cancer [10] and activation of Stat3 is stimulated by cytokines and growth factors [11]. Stat3 is crucial in normal development with embryonic lethality in Stat3 null mice [12]. Mammary-specific deletion of Stat3 has been shown to significantly delay involution [13, 14]. In cancer, Stat3 contributes to proliferation, apoptosis [15], and angiogenesis [16]. In HER2 induced mouse model tumors, Stat3 was noted to coordinate metastatic progression of the tumors [17]. Stat3 also stimulates immune responses in tumor microenvironment [18]. Impaired immune responses in MMTV-PyMT tumors without Stat3 led to defective metastasis [19]. In addition, vascular endothelial growth factor (VEGF) upregulates expression of Myc and Sox2 via Stat3 activation in tumor-initiating stem cells [20].

Given the functions of Stat3 and widespread effects of Myc in breast cancer, it is therefore essential to examine the interaction of these two genes *in vivo*. To test the hypothesis that Stat3 alters Myc tumorigenesis, we interbred MMTV-Myc mice with a conditional knockout of Stat3. This revealed unexpected roles for Stat3 in tumor development but not metastasis.

## RESULTS

### Stat3 has diverse functions in MMTV-Myc and MMTV-Neu tumors

**Figure 1:**
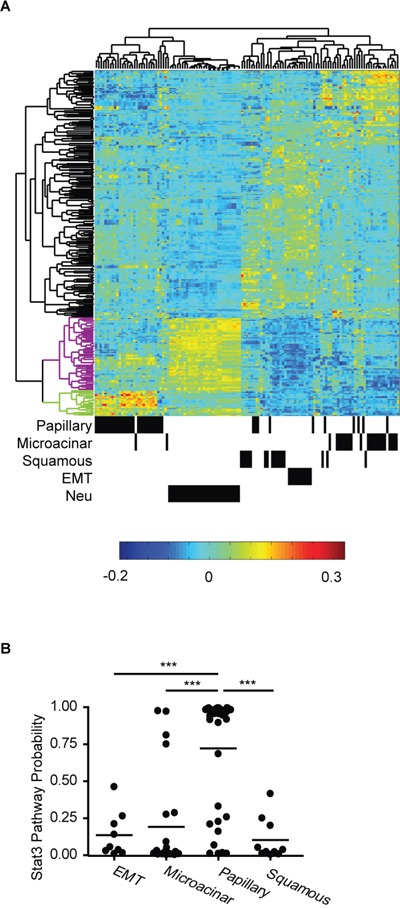
Stat3 has diverse functions in MMTV-Myc and MMTV-Neu tumors **A.** Unsupervised clustering of Stat3 pathway genes in MMTV-Myc and MMTV-Neu tumors is shown. The dendrogram on the top shows the relationship among the individual tumors. The annotated information for histological subtypes of MMTV-Myc and MMTV-Neu tumors is shown at the bottom where a black bar represents a tumor sample. The vertical dendrogram represents clusters of genes. Black clusters represent genes downregulated by Stat3 activation. Purple and green clusters include genes up-regulated by Stat3 activation in MMTV-Neu tumor and in Myc papillary subtype tumors respectively. **B.** Probability of Stat3 pathway activation in subtypes of MMTV-Myc tumors. ***p<0.001, two-tailed Student's t test.

To investigate the role of Stat3 in different oncogene-driven tumors, we compared gene expression profiles of MMTV-Myc and MMTV-Neu tumors. First, significant up-regulated and down-regulated genes from the Stat3 pathway [21] were identified. Gene expression from Myc and Neu induced tumors were then filtered to this list of Stat3 pathway genes and were used in unsupervised clustering. As shown in Figure 1A, MMTV-Neu tumors, and the various subtypes of Myc induced tumors were clustered into histological groups. Analysis of Stat3 pathway genes enriched in MMTV-Neu tumors revealed that many of these genes are associated with metastasis. In contrast, the Stat3 pathway genes enriched in papillary tumors were not able to stratify patients for metastasis effects (data not shown). These data indicate that Stat3 might have differential roles in MMTV-Myc and MMTV-Neu induced tumors. To test the role of the Stat3 genes in a predictive manner, we used the Stat3 pathway signature to test for activation in subtypes of MMTV-Myc tumors. Using the Stat3 signature [21, 22] in a binary regression approach [23], we predicted Stat3 activity in Myc induced tumors. Compared to other subtypes, the majority of papillary tumors had elevated Stat3 pathway probability (Figure 1B). Taken together, these data suggest the hypothesis that Stat3 has various roles in specific subtypes of MMTV-Myc tumors and that these roles are different from those previously observed in MMTV-Neu tumors.

### Roles of STAT3 in physiological functions

**Figure 2:**
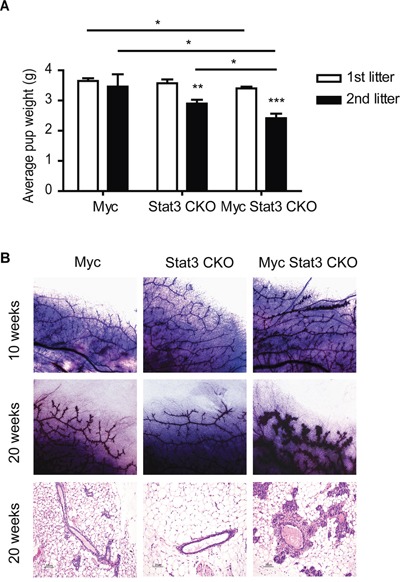
Physiological effect of STAT3 loss on mammary function **A.** Effects of STAT3 loss on the body weight of pups nursed by different genotypes of dams including MMTV-Myc, Stat3 CKO and Myc Stat3 CKO. The litter size was normalized to six pups one day post-partum. The average pup body weights of the first and second litters on Day 5 are shown with an n of at least six per group. Data were analyzed by two-tailed t-test. * p<0.05; **p<0.01; ***p<0.001. **B.** Whole mount staining (top and middle rows) in 10- and 20-week-old virgin mice as well as hematoxylin and eosin staining (bottom row) of mammary glands in 20-week-old virgin mice are shown. Representative images were chosen for each.

To determine the role of Stat3 in MMTV-Myc tumors, we interbred MMTV-Myc, MMTV-Cre, and Stat3^Fl/Fl^ mice to generate Stat3 conditional knockout mice in the MMTV-Myc background (Myc Stat3 CKO). We noticed that pups from the Myc Stat3 CKO dams were runted or died before weaning age. These pups were fully rescued with foster dams, demonstrating that vitality was due to defects in the Myc Stat3 CKO dams. To study the effects of Stat3 in normal developmental functions with Myc overexpression, we monitored the first litters of Myc Stat3 CKO, Stat3 CKO, and Myc dams. Litter size was normalized to 6 at postnatal day 1 and pup weight was tracked (Figure 2A). For the first and second litters of each group we noted a significant reduction in average weight from the Myc transgenic dams lacking Stat3. For the first litter, control Myc transgenic dams reared pups with an average weight of 3.7 grams at 5 days postpartum. There was a significant reduction in the Myc transgenic mice lacking Stat3 to 3.4 grams. In the second litter, this difference was more pronounced with a reduction to 2.4 grams in the Myc Stat3 CKO dams. Indeed, many pups from Myc Stat3 CKO dams did not have any visible milk spots during postpartum days 1-5. In the third litter of this group, several pups from Myc Stat3 CKO dams died before day 5. These data demonstrated that deletion of Stat3 is associated with the lower pup body weight, and the presence of the Myc transgene exacerbates this defect and suggested that Stat3 might have other roles in normal mammary gland development.

To examine the role of Stat3 in Myc transgenic mammary glands, we compared morphology changes in 10 and 20 week-old virgin mice using whole mount staining and histology (Figure 2B). At both 10 and 20 weeks, Myc transgenics had more side branches than their Stat3 CKO counterparts. Interestingly, the Myc Stat3 CKO mice had progressed to hyperplasia by 20 weeks of development. The histology of these various strains was consistent with the whole mounts. Taken together, these results suggest that loss of Stat3 reduced body weight of their pups, and expedites hyperplasia in the transgenic Myc background.

### Loss of STAT3 alters tumor onset, tumor growth and histology

**Figure 3:**
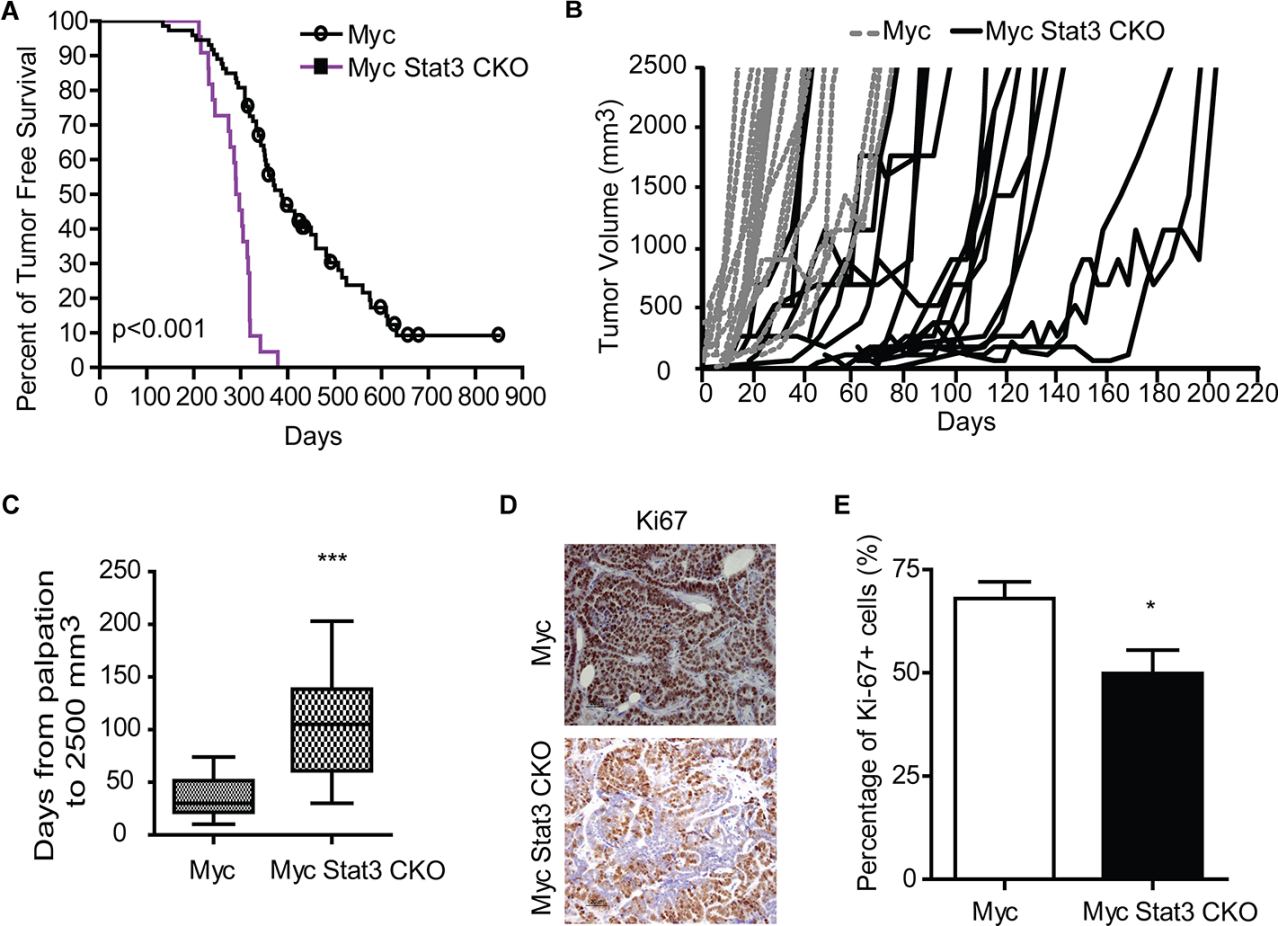
Loss of STAT3 alters tumor onset and tumor growth **A.** A Kaplan-Meier plot of tumor-free survival for MMTV-Myc (n=73) and Myc Stat3 CKO (n=22) is shown. **B.** Tumor growth curves are shown for individual tumors. MMTV-Myc mice are shown with dashed lines (n=20). Myc Stat3 CKO mice are shown with solid black lines (n=18). **C.** A box plot of days from palpation to 2500 mm^3^ reveals slowed tumor growth in the Myc Stat3 CKO mice with an average of 36 days from palpation to endpoint in MMTV-Myc mice (n=32), and 109 days in the Myc Stat3 CKO mice (n=20). ***p<0.001. **D.** Ki67 staining of mammary tumors in MMTV-Myc and Myc Stat3 CKO mice reveals a reduction in Ki67 staining. Representative pictures (D) and quantification results **E.** are shown. Data are mean ± S.D.*p<0.05. ***p<0.001, two-tailed Student's t test.

Given the hyperplastic phenotype in the Myc Stat3 CKO mice, we investigated if loss of Stat3 in MMTV-Myc mice accelerated tumor formation. Consistent with the mammary gland histological findings, Myc Stat3 CKO mice developed mammary tumors significantly more quickly than Myc transgenics (p<0.001). Median tumor onset was 294 days in Myc Stat3 CKO mice and 360 days in Myc mice with Stat3. Interestingly, we noted that once tumors were palpable in the Myc Stat3 CKO mice, the majority of tumors grew relatively slowly compared to the Myc control tumors (Figure 3B). Measuring the time from initial palpation to 2500 mm^3^ in Myc, and Myc Stat3 CKO mice revealed an average of 36 and 109 days respectively (Figure 3C). Consistent with this, Ki67 staining results supported the observation that tumors in Myc mice proliferated faster than those in Myc Stat3 CKO mice (Figure 3D and 3E, p=0.04).

**Figure 4:**
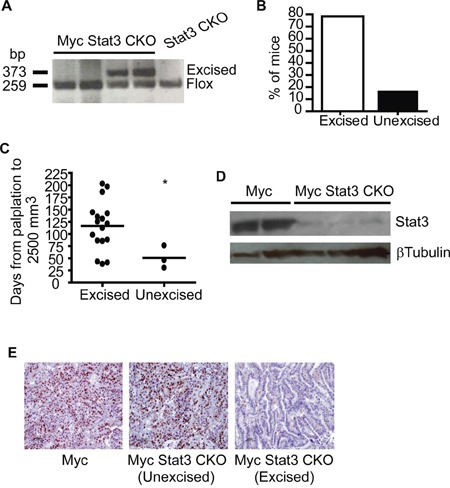
Incomplete excision of Stat3 in mammary tumors **A.** Excision of Stat3 in tumors was tested using PCR with tail DNA of STAT3^Fl/Fl^ mice as a control. **B.** Excision PCR demonstrated that 16% (6 out of 37) tumors did not have detectable STAT3 excision. **C.** Examination of tumor growth rate showed that excision of Stat3 in Myc induced tumors was associated with delayed growth rate. *p= 0.04, two-tailed Student's t test. **D.** To ensure excision was associated with protein loss, a Western blot for STAT3 in mammary tumors from MMTV-Myc and Myc with excised Stat3 was conducted. **E.** Representative images are shown from immunohistochemistry for pSTAT3 (Y705) conducted on Myc tumors and Myc Stat3 CKO tumors without and with excision.

While the delay in growth rate was substantial with loss of Stat3, we noted that several tumors that appeared to grow at the same rate as the Myc control tumors. Given the previous observation that tumors with MMTV-Cre directed excision could select against tumor progenitor cells with Cre expression [24], we examined tumors from Myc Stat3 CKO mice for excision. PCR on primary tumors to test for Stat3 excision revealed a number of tumors with and without excision (Figure 4A). Indeed, of the 38 tumors examined, 10 lacked Stat3 excision (Figure 4B). When correlated with tumor growth of Myc Stat3 CKO mice, those tumors lacking Stat 3 excision had more rapid tumor growth relative to tumors with Stat3 excision (Figure 4C). At the same time, protein expression of Stat3 from tumors with Stat3 excision was tested, confirming the knockout (Figure 4D). While there is a weak signal in the excised tumor samples, it is important to note that the MMTV-Cre transgene only directs expression to the mammary epithelial cells and that there are a number of additional cell types in a tumor. As a further confirmation of functional excision, we performed immunohistochemical staining of phosphorylated Stat3 (Tyrosine 705) to detect the active form of Stat3. As shown in Figure 4E, tumors in Myc and unexcised-Stat3 tumors in Myc Stat3 CKO mice had strong pStat3 signals. Importantly, Stat3-excised tumors in Myc Stat3 CKO mice did not have detectable pStat3.

**Figure 5:**
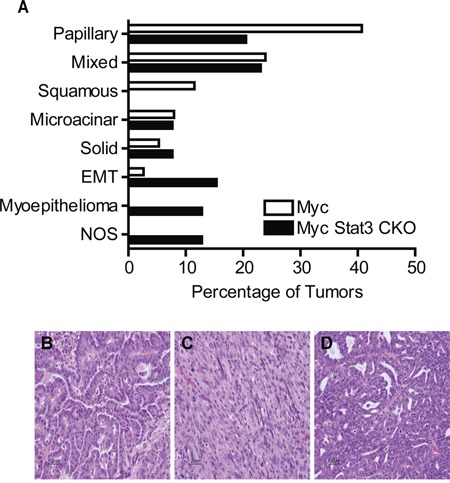
Loss of Stat3 alters tumor histology **A.** Quantification of the histological subtypes of mammary tumors observed in MMTV-Myc and Myc Stat3 CKO mice revealed histological differences in Myc induced tumors lacking Stat3. EMT – Epithelial to Mesenchymal Transition, NOS – Not otherwise specified. Representative images with hematoxylin and eosin staining from various subtypes including papillary **B.** EMT **C.** and myoepithelioma **D.** are shown.

Given the alterations to growth rate of the tumor and the selective pressure to maintain Stat3 expression, we hypothesized that loss of Stat3 may result in alterations to the morphology of the tumors. After a histological examination we noted changes in subtypes of these tumors (Figure 5A). Notably, 40% of control MMTV-Myc tumors typically have a papillary pattern (Figure 5A and 5B). With the loss of Stat3, this was reduced to 20.5%. In addition, MMTV-Myc tumors frequently developed a squamous pathology, readily detected through the accumulation of keratin pearls. Interestingly, this subtype was not detected in tumors lacking Stat3 (Figure 5A). Moreover, the EMT tumors that were detected at a low frequency in the Myc transgenics (2.7%) were significantly enriched (15.4%) with loss of Stat3 (Figure 5A and 5C). In addition, we noted an increase in myoepithelioma after loss of Stat3 in these tumors (Figure 5A and 5D). Together, these results indicate that loss of Stat3 resulted in significant histological alterations to the Myc induced tumors in addition to the acceleration of tumor initiation and decreased tumor growth rate.

### STAT3 ablation impairs angiogenesis and inflammation

**Figure 6:**
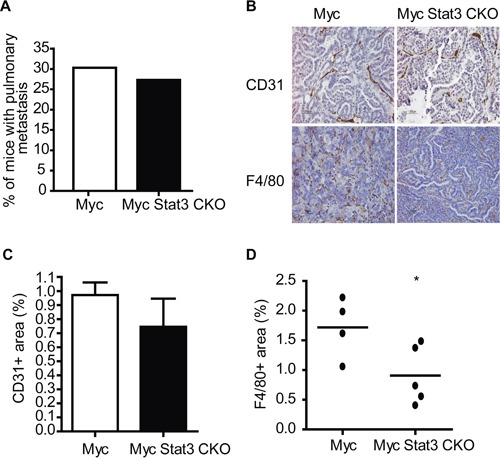
STAT3 ablation impairs angiogenesis and inflammation **A.** The percentage of mice with lung metastases at end point (tumor volume of 2500 mm^3^) did not reveal an alteration in metastatic capacity with loss of Stat3. **B.** Immunohistochemistry for CD31 in mammary tumors from MMTV-Myc and Myc Stat3 CKO mice reveals a decrease in CD31 staining. **C.** Quantification of CD31 staining was determined by CD31+ per tumor area (mm^3^). n=5 per genotype and n=5 per mouse. Data are mean ± S.D. **D.** F4/80 staining of mammary tumors in MMTV-Myc and Myc Stat3 CKO mice revealed a reduction in F4/80 staining with the loss of Stat3. The percentage of F4/80 was calculated by F4/80+ per tumor area (mm^3^) and quantification is shown *p<0.05.

With the increase of the EMT subtype and given the previously identified role of Stat3 in breast cancer metastasis [17], we investigated how loss of Stat3 altered metastasis in the MMTV-Myc strain. Surprisingly, we noted that there was not a significant difference in tumor bearing mice with pulmonary metastasis when mice reached the endpoint of 2500 mm^3^ (Figure 6A). In addition, we noted no differences in the size or number of metastatic lesions in the lung. While there were no significant differences, it is important to consider that the alterations to growth rate in the Myc Stat3 CKO mice resulted in tumors that were in the mouse for over twice the length of time of MMTV-Myc controls and it is therefore possible that Stat3 does reduce metastasis.

To test this premise, we examined the consequence of Stat3 loss on tumor vasculature. Through CD31 staining, we observed that tumors with Stat3 excision have less vessels than those with endogenous Stat3 (Figure 6B top panel). After quantification, tumors lacking Stat3 were noted to be trending towards lower CD31-positive signals than those with Stat3 (Figure 6C). Next we examined if tumors without Stat3 in the Myc transgenic background had altered inflammation. As predicted, F4/80 staining revealed tumors in MMTV-Myc mice have more macrophages than those in Myc Stat3 CKO mice (Figure 6B bottom panel and 6D). Taken together, these results suggest that Stat3 reduced both angiogenesis and inflammation in My induced tumors.

## DISCUSSION

The heterogeneity of breast cancer has been well characterized at the gene expression level, leading to the development of numerous classes of breast cancer [1, 2]. The heterogeneity is also reflected in the cell signaling pathways that are activated in tumor samples in both human breast cancer and mouse models [22, 25, 26]. Here we have used a bioinformatic method to predict a role for Stat3 in Myc induced tumors. Myc and Stat3 have been found to be associated with the resistance to both endocrine therapy [27] and chemotherapy [15]. In addition, their interaction has been noted in tumor-initiating cells resistant to chemotherapy [20], underscoring the importance of understanding the underlying biology.

To decipher the role of Stat3 in Myc tumors, we generated a mammary specific knockout of Stat3 in MMTV-Myc transgenic mice. Unexpectedly, we noted mammary gland effects during lactation. Pups nursed by a Myc Stat3 CKO dam grew abnormally slowly or died. Growth of these pups was similar to other pups after being fostered by dams of other genotypes. While deletion of Stat3 in the mammary gland has been described to delay involution [13, 14, 28], we were surprised to note the lactation defects. However, mice lacking Socs3, a negative regulator of Stat3, have been found with precocious involution due to increased apoptosis with elevated expression of Myc and Stat3 [29]. Similarly, overexpression of Myc resulted in accelerated lactation, and earlier onset of involution during pregnancy through activation of Stat3 [30]. Together these results suggest that deletion of Stat3 with Myc overexpression leads to lactation deficiencies.

In comparison to control MMTV-Myc mice, loss of Stat3 increased the formation of hyperplastic areas in the mammary glands, accelerated tumorigenesis, and slowed tumor growth. MMTV-Myc mice were initially reported to spontaneously develop adenocarcinomas [6] and further studies revealed that additional mutations, such as Kras, were accumulated in Myc-driven tumors [31]. Stat3 has been shown to sensitize cells to DNA damage [32]. Thus, it is plausible that Stat3 activates DNA repair pathways to repair DNA damage caused by Myc, thereby delaying tumor onset. Without Stat3, these tumorigenic cells are able to continuously grow, resulting in the accelerated tumor detection that we described. During proliferation, Myc is known to shorten G1 phase and accelerate the cell cycle [3], and Stat3 could be activated after stimulation of growth factor through Janus Kinase 2 (JAK2). Consistent with our findings, expression of dominant-negative Stat3 or treatment with a JAK2/Stat3 inhibitor in breast cancer cell lines inhibited cell growth [33].

In contrast to other mouse model studies, Myc / Stat3 interactions were noted to be unique. For instance, loss of Stat3 did not alter tumor onset of Neu induced tumors, and led to significantly reduced metastasis [17]. In the highly metastatic PyMT model, ablation of Stat3 also abrogated metastasis [34]. In contrast, we did not observe significant metastatic effects in the Myc background. These data indicate that Stat3 has a tumor specific context for the metastatic role. Recent studies also support the theory that Stat3 acts as an oncogene as well as a tumor suppressor depending on the oncogene-driven context. Constitutive activated Stat3 blocked the transformation of mouse embryonic fibroblast triggered by Myc, but did not suppress transformation driven by Harvey rat sarcoma viral oncogene homolog (Hras) [35]. Moreover, lack of Stat3 in Kras induced lung adenocarcinoma accelerated tumorigenesis [36].

Taken together, our data have revealed a role for Stat3 in normal and tumor development as well as tumor progression. These findings demonstrate a role for Stat3 that is unique to Myc induced tumors when compared to other oncogenic models. These findings suggest that extrapolation to a role for Stat3 in human cancer should be carefully considered for driving mutations inducing breast cancer.

## MATERIALS AND METHODS

### Mouse dataset and bioinformatics analysis

Microarray data of MMTV-Myc and MMTV-Neu tumors were from GSE 15904 [25]. Significant analysis of microarrays (SAM) [37] was used to select signature genes from Stat3 signature expression data [21]. Unsupervised clustering was generated using Cluster 3.0 and Java Treeview. Probability of Stat3 pathway activity was generated as described [22, 25].

### Animals

Stat3^Fl/Fl^ MMTV-Cre MMTV-Myc and Stat3^Fl/Fl^ MMTV-Cre mice were generated from interbreeding MMTV-Myc [25], MMTV-Cre [38], and Stat3^Fl/Fl^ mice generated in the Levy laboratory [14, 39]. Stat3^Fl/WT^ mice and MMTV-Cre mice were a generous gift from Dr. William J Muller at McGill University. Body weight of pups was measured daily. Tumors were monitored at least twice per week, and were measured using a caliper. Tumor volume was calculated as the shortest diameter^2^ × the longest diameter/2. All mice were bred and maintained according to guidelines and protocols approved by the Institutional Animal Care and Use Committee in Michigan State University.

### Wholemount staining, immunohistochemistry staining, and immunoblotting

Wholemount staining was performed on the mammary glands of virgin mice using Harris's modified hematoxylin. Paraffin-embedded sections were used to perform immunohistochemical analyses. Ki-67 antibody (Ab15580) was from Abcam (Cambridge, MA, USA). F4/80 antibody (Q61549) was from Serotec (Oxford, United Kingdom). CD31 antibody (DIA-310) was from HistoBioTec (Miami beach, FL, USA). Stat3 (#9139), phospho-Stat3 (Tyr705, #9145), and beta-tubulin (#2128) antibodies were purchased from Cell Signaling Technology (Beverly, MA, USA). Secondary goat anti-mouse and secondary goat anti-rabbit antibodies were from Abcam. Positive signals were quantified using Image J software.

### PCR primers

The primer sequences used in determining Stat3 excision were; Stat3^WT/Fl^(1): 5′-GCTGCCAACAGCCACTGCCCCAG-3′;Stat3^WT/Fl^ (2): 5′ GAAGGCAGGTCTCTCTGGTGCTTC-3′;Stat3 ko: 5′-CAGAACCAGGCGGCTCGTGGCG-3′.

### Statistical analysis

Analysis was completed with Graphpad prism.
